# Intersection of Big Five Personality Traits and Substance Use on Social Media Discourse: AI-Powered Observational Study

**DOI:** 10.2196/79454

**Published:** 2025-12-19

**Authors:** Julina Maharjan, Ruoming Jin, Jianfeng Zhu, Deric Kenne

**Affiliations:** 1 Department of Computer Science, Kent State University, 800 East Summit Street, Kent, OH, 44242, United States, 1 3305931365; 2 Department of Public Health, Kent State University, Kent, OH, United States

**Keywords:** agreeableness, extraversion, neuroticism, openness, conscientiousness, Big Five personality traits, substance use, natural language processing, NLP, deep learning, social media, X, COVID-19

## Abstract

**Background:**

Personality traits are known predictors of substance use (SU), but their expression and association with SU in digital discourse remain largely unexamined. During the COVID-19 pandemic, the online social engagement heightened and led to an amplification in SU rates, thereby creating a unique natural opportunity to investigate these dynamics through large-scale digital discourse data. In our study, we offer insights beyond traditional self-report methods, which are crucial for developing timely and targeted public health interventions.

**Objective:**

We aim to evaluate whether the associations between the Big Five personality traits and SU discourse shifted during the 2019‐2021 period, and to conduct a focused analysis of how these traits predict SU and relate to specific substance types, emotional expression, and demographic factors.

**Methods:**

We analyzed a corpus of several hundred million public posts from a major social media platform from 2019 to 2021. Using a pipeline of natural language processing and deep learning models, we identified SU-related posts and subsequently extracted scores for the Big Five personality traits, emotions, and user demographics. We used trend analysis to compare annual shifts in trait-SU associations, while detailed 2020 data underwent rigorous modeling using logistic regression, correlation analysis, and topic modeling to elucidate the core relationships.

**Results:**

Our analysis revealed that Extraversion (odds ratio [OR] 3.22, 95% CI 2.98-3.49) and, most strikingly, agreeableness (OR 4.04, 95% CI 3.71-4.41) were the strongest positive predictors of being a substance user. In stark contrast to the conventional self-medication hypothesis, neuroticism emerged as a robust protective factor against SU (OR 0.29, 95% CI 0.26-0.31). This counterintuitive finding was supported by a decreased association between neuroticism and SU posts at the pandemic’s onset in 2020 (Cohen *d*=−0.13, 95% CI) and a negative correlation with the expression of negative emotions online. Topic modeling further indicated that SU discourse was frequently embedded in social contexts (social drinking and friendly beverage choices) rather than themes of solitary coping.

**Conclusions:**

Our findings challenge traditional models by demonstrating that in large-scale online discourse, SU expression is more powerfully linked to social-affiliative traits than to negative emotionality. The paradoxical protective role of neuroticism suggests that established risk profiles may not apply uniformly to digital environments, particularly during a public health crisis. These insights are vital for refining computational methods for public health surveillance and developing interventions that recognize the potent social drivers of SU in the digital age.

## Introduction

### Background

Personality traits are established predictors of substance use (SU), shaping both vulnerability to and protection from risky behaviors [1,2]. The Five-Factor Model of personality, which defines personality through the core dimensions of openness, conscientiousness, extraversion, agreeableness, and neuroticism, provides a robust framework for this purpose [3]. The brief description that characterizes each trait, along with the dimension intended to measure, is highlighted in Textbox 1.

For decades, a rich body of research has established foundational links between these personality traits and risky health behaviors, including smoking, excessive alcohol consumption, and poor dietary choices, with high neuroticism and low conscientiousness consistently emerging as key vulnerability factors [4]. Within this broader behavioral landscape, these traits also shape vulnerability to SU, influencing both initiation and maintenance of risky consumption patterns [5-8]. The association between personality and SU is not monolithic; specific traits consistently emerge as powerful risk or protective factors. High neuroticism, the predisposition to negative emotions, is a robust predictor of SU, as individuals may turn to substances to self-medicate or manage pervasive anxiety [6]. Conversely, high conscientiousness, characterized by prudence and self-discipline, is a significant protective factor against the impulsivity inherent in substance misuse [8]. The roles of other traits are more nuanced: the reward-seeking facets of extraversion can increase initiation in social settings, the curiosity of openness can drive experimentation, and the antagonism of low agreeableness can foster risk through nonconformity [5,9].

Textbox 1 **. Overview of Big Five personality traits.**

Openness: represents an individual’s level of curiosity, imagination, and willingness to try new things.

Conscientiousness: reflects how organized, responsible, and detail-oriented an individual is.

Extraversion: indicates how outgoing, sociable, and energetic an individual is.

Agreeableness: measures how cooperative, trusting, and kind an individual is.

Neuroticism: represents the level of emotional instability, anxiety, and mood swings an individual experiences.

At the same time, personality expression on social media is shaped by norms of digital self-presentation and impression management. Individuals often adapt their communication to maintain a favorable or socially desirable image in public online spaces. This framing is particularly relevant for traits such as agreeableness, which may manifest as affiliative, communal, or prosocial discourse even when discussing behaviors such as drinking or drug use. Thus, the personality-SU link on social media may reflect not only underlying psychological dispositions but also the performative dimensions of digital interaction.

Against this backdrop, major societal disruptions provide unique opportunities to observe how established trait-behavior relationships may shift under conditions of collective stress. The COVID-19 pandemic represents one such exogenous shock: while no longer the central motivation of this study, it created an unparalleled context in which SU behaviors and digital expression were amplified [10,11]. The pandemic functioned as a natural experiment for several reasons. First, it imposed sudden, widespread changes to daily routines, social interaction, and stress levels across diverse populations, creating variation in environmental conditions that could influence SU behaviors. Second, it triggered a simultaneous increase in online social engagement, producing rich, time-stamped digital traces that allowed for the observation of behavior in near real time. Third, because the event was unplanned and affected populations broadly, it reduced potential confounding from self-selection into specific contexts, providing a rare opportunity to examine how personality traits interact with situational stressors to shape SU discourse. Enforced isolation, for example, may have transformed extraversion from a facilitator of recreational use into a risk factor for coping with loneliness [11]. Situations of this magnitude challenge traditional research methodologies, as controlled experiments or surveys often lack ecological validity to capture authentic behavioral expressions under rapidly shifting societal conditions [12-14].

This methodological gap is particularly salient given the surge in digital communication during the pandemic. Platforms such as Twitter (subsequently rebranded X; X Corp) became central arenas for processing the crisis, creating rich new sources of naturalistic behavioral data. This development has coincided with advances in computational social science, which enable the assessment of psychological states, including personality, through the analysis of natural language at a scale and temporal precision previously unimaginable [15]. These digital phenotyping approaches capture authentic linguistic markers of personality in real-world contexts, offering an unprecedented opportunity to examine how the relationship between dispositional traits and SU discourse was reshaped by the pandemic.

### Literature Review

#### Big Five Personality Traits and Their Relationship With SU

A robust consensus exists regarding the general relationship between the Big Five personality dimensions and SU [2]. A “problem-behavior” profile consistently emerges from meta-analyses and large-scale studies, centrally featuring high neuroticism and low conscientiousness. Neuroticism, the tendency to experience negative emotions such as anxiety and depression, is a powerful predictor of both SU initiation and the development of substance use disorders (SUDs). The prevailing self-medication hypothesis posits that individuals high in this trait turn to substances to cope with or temporarily alleviate their emotional distress [6,16]. Complementing this, low conscientiousness, characterized by impulsivity, poor planning, and a deficit in self-discipline, is strongly linked to a general propensity for risky health behaviors, including the misuse of a wide range of substances [8,17].

The roles of the other major traits are more nuanced. High extraversion, particularly its facet of sensation-seeking, is often associated with the initiation of recreational SU in social contexts, driven by a desire for stimulation and positive affect. Openness to experience, which reflects intellectual curiosity and a preference for novelty, is frequently linked to experimentation with drugs, especially cannabis and hallucinogens. Finally, low agreeableness, which involves antagonism and nonconformity, can increase SU risk through pathways of social deviance and a disregard for prohibitive social norms [15,18].

#### Specificity in Trait-Drug Relationships

Beyond a general vulnerability, research increasingly reveals that specific traits predispose individuals to use particular classes of drugs, aligning with distinct motivational pathways. For instance, the strong link between neuroticism and SU is most pronounced for depressants such as alcohol and opioids, which are perceived to dampen anxiety and align with a negative reinforcement or “coping” model of use [19,20]. In contrast, extraversion is more directly predictive of stimulant use such as cocaine and amphetamines, which enhances social energy and reward, reflecting a positive reinforcement pathway [21]. The established connection between openness and the use of cannabis and psychedelics is often driven by motives related to cognitive and perceptual expansion rather than social facilitation or distress-coping [15]. This evidence suggests that personality does not merely predict if someone will use substances, but often what they will use and why.

#### The Moderating Role of Demographics: Age and Gender

The strength and expression of these personality-SU relationships are further moderated by key demographic factors. Age is a critical variable, as the influence of certain traits evolves across the lifespan. The impact of impulsivity and sensation-seeking (linked to low conscientiousness and high extraversion) is most powerful during adolescence and young adulthood in predicting the initiation of SU [22]. In later adulthood, however, traits such as neuroticism may become more salient predictors of the maintenance or escalation of SU as a chronic coping strategy for life stressors [8].

Gender also shapes these pathways [23,24]. While men have historically shown higher rates of SUDs, women often exhibit a “telescoping” effect—a more rapid progression from initial use to dependence. Research suggests the link between neuroticism and coping-motivated SU is particularly strong in women, who may be more likely to use substances to self-medicate for co-occurring mood or anxiety disorders. Conversely, SU pathways related to impulsivity and social deviance (low conscientiousness and low agreeableness) appear to be more pronounced predictors for men [12].

#### Personality Assessment in the Digital Era: Social Media, Personality, and SU

Despite these well-documented associations, traditional trait-SU research has been constrained by its reliance on self-report surveys and controlled experiments. These methods, while valuable, often suffer from recall bias, social desirability effects, and a lack of ecological validity, failing to capture authentic behavioral expressions in real-world contexts. The proliferation of social media has created an unprecedented opportunity to overcome these limitations [25]. Platforms such as Twitter serve as vast, naturalistic archives of human thought and behavior where individuals openly discuss their experiences, including SU.

The emerging field of digital phenotyping leverages computational methods, particularly natural language processing (NLP), to infer psychological traits and monitor health behaviors from user-generated digital footprints [26]. Recent studies have successfully demonstrated the power of these approaches in capturing nuanced behavioral and personality patterns: AI-based analysis of social media language can predict SY treatment outcomes [27], while cognition-aware digital phenotyping frameworks provide fine-grained monitoring of SU behaviors in real-time [28]. Moreover, digital phenotyping is increasingly recognized as a transformative tool in psychiatry, offering data-driven insights into mental health, including SU risk, and facilitating ecologically valid assessments of personality-SU interactions [4,29].

This methodology allows researchers to study the personality-SU link at a massive scale, with temporal precision, and within the authentic context of users’ daily lives, providing a powerful complement to traditional approaches. However, the application of these methods to examine how a global stressor such as the COVID-19 pandemic reshaped the public discourse around personality and SU remains a significant and underexplored frontier.

This study addresses these research gaps by applying advanced computational methods to a massive longitudinal dataset of 1.13 billion tweets spanning the prepandemic and pandemic periods (2019‐2021). By leveraging deep learning-based personality inference and state-of-the-art topic and emotion modeling, we provide a multidimensional examination of how personality traits manifested in natural SU discourse. Our study aims to make 3 primary contributions: first, to establish the utility of large-scale social media data for studying personality-SU relationships in ecologically valid contexts; second, to reveal how the crisis conditions of the COVID-19 pandemic modified these associations; and third, to demonstrate the value of computational methods for identifying at-risk populations and informing targeted public health interventions. To achieve these aims, this study is guided by the following research questions (RQs) in Textbox 2.

Textbox 2 **. Research questions (RQ).**

RQ1: longitudinal variation: did the linguistic expression of Big Five personality traits within substance use (SU)-related discourse on Twitter (subsequently rebranded X; X Corp) exhibit significant variation between the prepandemic (2019) and pandemic (2020-2021) periods?

RQ2: predictive analysis: to what extent do the Big Five personality traits predict SU classification among individuals on the platform Twitter during the unique socioenvironmental context of the COVID-19 pandemic?

RQ3: emotional expression: how did the emotional tone of SU-related discourse vary across individuals with different dominant personality traits throughout the pandemic?

RQ4: substance specificity: what were the associations between inferred personality traits and discourse related to specific substance types (eg, alcohol, cannabis, or opioids)?

RQ5: demographic dimensions: how did the relationships between personality and SU discourse differ across key demographic factors, specifically age and gender?

RQ6: thematic content: what were the prevailing topics within SU discourse, and how did these themes differ across the 5 major personality traits during the pandemic?

## Methods

### Overview

**Figure 1.**
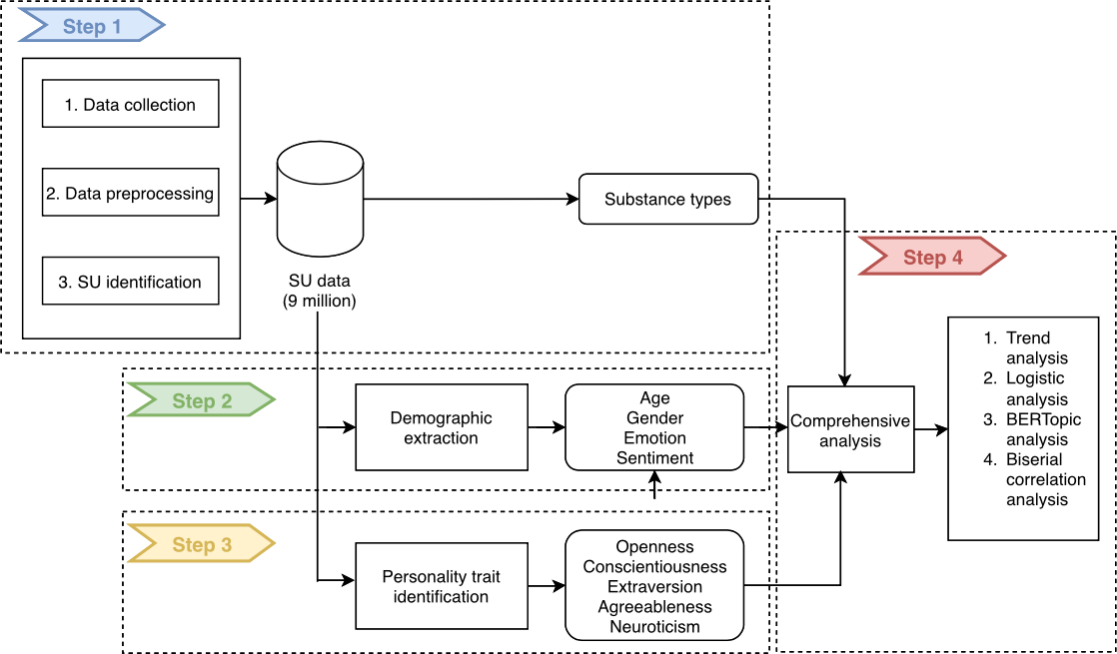
Overview of research work. SU: substance use.

The methodology for this study is systematically organized into 4 stages, as depicted in Figure 1. This section details each stage, beginning with the construction of the SU data corpus (step 1), followed by the parallel extraction of demographic or emotional attributes (step 2) and personality traits (step 3). These streams converge into the final stage, a comprehensive statistical analysis (step 4), designed to address the study’s RQs. The reporting of this observational study adhered to the STROBE (Strengthening the Reporting of Observational Studies in Epidemiology) guidelines [30], and a completed STROBE checklist detailing the study design and methods is provided in Checklist 1.

### Data Acquisition and SU Identification (Step 1)

The foundation of this study is a large-scale data corpus constructed from the Internet Archive’s public Twitter stream [31], spanning January 1, 2019, to December 31, 2021. This 3-year period provides a critical prepandemic baseline (2019) for comparison against the pandemic’s subsequent phases (2020‐2021).

The process began with data collection and extensive data preprocessing. We filtered the raw data to retain US-based, original posts (excluding retweets), tweets shorter than 5 words, and applied a rigorous cleaning pipeline using the Natural Language Toolkit. This involved converting text to lowercase, removing punctuation, stop words, URLs, and user mentions, and performing lemmatization to standardize words to their base forms (eg, “drinking” to “drink”).

The cleaned corpus of 1.13 billion posts was then processed for SU identification. We used a deep learning classifier from our prior work [32], which uses a RoBERTa [32] architecture to identify SU-related discourse with 80% accuracy. This step yielded the final SU data corpus of approximately 9 million posts. As the final part of this stage, we further classified these posts into 10 substance types based on the National Institute on Drug Abuse’s pharmacological categories [33] (alcohol, tobacco, cannabinoids, stimulants, opioids, hallucinogens, club drugs, dissociative drugs, other compounds, and prescription medication) using standard keywords formulated by National Institute on Drug Abuse. For instance, the post is identified as tobacco only if keywords associated with either one of cigarettes, vapor cigarettes, cigars, chewing tobacco, and snuff are present in the post. These subcategories of classification ensured the foundational data for all subsequent analyses.

### Demographic and Emotional Feature Extraction (Step 2)

Taking the 9 million post SU data corpus as input, this stage focused on extracting user-level demographic and post level emotional attributes [34].

### Demographic Extraction

We inferred user age and gender using the M3-Inference model [35], an open-source system that analyzes textual features from user profiles (username, screen name, and description). As validated against ground-truth profiles, the model achieved high accuracy for the gender (95%) and age (89%) classification. Age was categorized into 4 groups (≤18, 19‐29, 30‐39, and ≥40 years).

### Emotion and Sentiment Extraction

We analyzed the emotional content of each post using 2 tools. Multi-label emotion recognition was performed using SpanEmo [36], a BERT-based model that classifies text into 1 of 7 key emotions (fear, joy, negative, positive, sadness, surprise, and trust). For overall sentiment, we used VADER [37], a lexicon-based tool that calculates a single compound score ranging from −1 (most negative) to +1 (most positive).

### Big Five Personality Traits Identification (Step 3)

We derived users’ Big Five personality traits (openness, conscientiousness, extraversion, agreeableness, and neuroticism) from their social media posts using our validated deep learning model [38]. The model was trained on a Reddit dataset, PANDORA [39] comprising 1 million posts with professionally labeled Big Five personality scores. Our architecture used large language model embeddings (RoBERTa [40]) trained on a bidirectional long short-term memory network to build the personality trait classifier. Essentially, we build 5 classifiers for each trait in which we achieved an average of area under the receiver operating characteristic curve=0.8 across all traits. The performance of the classifier on each trait was 0.82 for openness, 0.81 for conscientiousness, 0.80 for extraversion, 0.83 for agreeableness, and 0.81 for neuroticism. Using an 8:1:1 data split for training, validation, and testing, the model achieved robust performance in personality trait prediction. As both training and application data consisted of social media content (Reddit and Twitter, respectively), we applied this model to estimate personality scores (range: 0‐1) for all SU-related posts in our dataset. This approach allowed for scalable personality assessment while maintaining consistency with established psychological measurement frameworks.

### Comprehensive Analysis (Step 4)

#### Trend Analysis

To explore temporal patterns in personality traits in SU discourse, we conducted a trend analysis examining the mean and SD of the continuous personality score (0 to 1) over the 3 years. The trend was further stratified by the age and gender variables.

#### Multilevel Logistic Regression

To examine temporal trends in the associations between Big Five personality traits and substance-related posts, we conducted logistic regression on the 2020 corpus. Standardized trait scores (mean 0, SD 1) were used as predictors, with substance types (binary: 0/1) as the outcome. Odds ratios (ORs) and 95% CIs were computed to quantify the strength and uncertainty of these associations. To visualize relational patterns, we plotted ORs with connecting lines, enabling clear comparison between multiple substances for a specific trait. Error bars represent 95% CIs, and a reference line at OR 1 indicates no effect. This approach allows for robust assessment of trait-specific substance patterns in social media discourse.

#### Biserial Correlation Analysis

The point-biserial correlation [41] is a special case of Pearson correlation used when 1 variable is dichotomous, and the other is continuous. It measures the relationship between a dichotomous (binary) variable and a continuous variable. In our study, we used it to determine the correlation between binary-valued substance type and a continuous Big Five personality score.

#### BERTopic Modeling

We implemented BERTopic [42] modeling to analyze SU discourse patterns across different personality traits, using a multistage pipeline that combined transformer-based embeddings with hierarchical clustering. The text was first encoded into 384-dimensional semantic vectors using SentenceTransformer’s “all-MiniLM-L6-v2” model, followed by UMAP (Uniform Manifold Approximation and Projection) dimensionality reduction (n_components=3, cosine metric) and HDBSCAN (Hierarchical Density-Based Spatial Clustering of Applications With Noise) clustering (min_cluster_size=50) to identify distinct topics. For topic representation, we used a hybrid approach incorporating KeyBERT for keyword extraction, Maximal Marginal Relevance (diversity=0.3) for term selection, and GPT-3.5 turbo for generating personality-specific topic labels through customized prompts. The custom prompt is presented in Textbox 3. While traditional topic models can identify clusters of semantically related words, their labels are often limited, ambiguous, or fail to capture subtle contextual nuances. GPT, in contrast, leverages deep contextual understanding to generate highly interpretable, human-readable, and personality-sensitive topic descriptors. This allows the model to capture the social, emotional, and behavioral subtleties embedded in SU discourse, providing richer and more actionable insights than conventional labeling methods.

Textbox 3 **. Large language model prompt for topic labeling.**

Prompt = """I have topic that contains the following documents: \n[DOCUMENTS]The topic is described by the following keywords: [KEYWORDS]Based on the above information, can you give a short label of the topic?"""

The model incorporated n-gram processing (1‐2 g) and filtered social media-specific stopwords while leveraging GPU acceleration for efficient computation, providing an optimized balance between semantic granularity and analytical efficiency for psychological text analysis.

### Ethical Considerations

This study was conducted in accordance with strict ethical protocols. The data was sourced exclusively from the publicly available Internet Archive, containing no private information. There was no exclusive participation accounted for in this research; hence, no compensation was made. A rigorous deidentification process, including the removal of user IDs and the preprocessing of post content, was implemented to ensure user anonymity. All findings are reported in aggregate to prevent the identification of individuals. To further mitigate ethical risks, this study framed its analyses to emphasize population-level insights, carefully contextualizing the associations between personality traits and SU behaviors to prevent misinterpretation or misuse in real-world applications. These measures were implemented throughout the research process to ensure responsible handling of potentially sensitive behavioral data.

Beyond these procedural safeguards, it is important to reflect on the ethical implications of inferring sensitive traits such as age, gender, personality, and SU behaviors without explicit consent, even from public data. Our analyses were intentionally designed to avoid individual-level profiling, prioritize transparency in modeling methods, and interpret findings at the population level. By acknowledging these concerns, we aim to promote the responsible, ethically informed use of computational social science methods and digital phenotyping in public health research.

This research was supported by the Substance Abuse and Mental Health Services Administration Strategic Prevention Framework-19 (grant 6H79SP081502) and was approved by the Institutional Review Board at Kent State University (IRB20-182).

## Results

### Descriptive and Trend Analysis in Personality Traits

This section presents the findings on the relationship between 5 major personality traits and SU trends observed annually from 2019 to 2021. For the identified SU posts (2,799,726 in 2019, 3,502,171 in 2020, and 2,553,235 in 2021) from our previous work [32], first, we predicted the score across each personality trait in a scale of 0 to 1 using our custom deep learning model [38]. The data, including mean scores, SDs, and effect sizes for year-over-year changes, are summarized in Table 1. The analysis reveals distinct patterns for each personality trait, with notable shifts occurring particularly in the year 2020, a period marked by the onset of the global COVID-19 pandemic.

**Table 1.** Personality traits and substance use trends (2019‐2021).

Trait	2019, mean (SD)	2020, mean (SD)	2021, mean (SD)	Trend interpretation^a^
Openness	0.47 (0.15)	0.49 (0.15)	0.47 (0.15)	↑ in 2020 (*d*=0.13), then stabilized
Conscientiousness	0.69 (0.13)	0.71 (0.12)	0.70 (0.13)	↑ in 2020 (*d*=0.15), slight ↓ in 2021
Agreeableness	0.38 (0.14)	0.39 (0.13)	0.39 (0.13)	Small ↑ in 2020 (*d*=0.07)
Neuroticism	0.63 (0.15)	0.61 (0.15)	0.61 (0.15)	↓ in 2020 (*d*=−0.13), contrary to expected pandemic stress effects
Extraversion	0.53 (0.14)	0.53 (0.14)	0.53 (0.14)	No change (*P*≥.99)

a Cohen *d* values (0.07-0.15) indicate effect size for the change from the previous year.

An examination of the data presented reveals distinct patterns for each personality trait across the 3 years. For openness, the association with SU showed a fluctuation. It initially stood at mean 0.47 (SD 0.15) in 2019, then increased in 2020 to mean 0.497 (SD 0.15). This rise represented a small effect size (d=0.13). Subsequently, in 2021, the association returned to its 2019 level of mean 0.47 (SD 0.15), indicating a stabilization. The association between conscientiousness and SU also shifted during this period. Starting at mean 0.69 (SD 0.13) in 2019, it increased to mean 0.71 (SD 0.12) in 2020, a change reflecting a small effect size (d=0.15). In 2021, there was a slight decrease in this association to mean 0.70 (SD 0.13). Agreeableness demonstrated a minor change in its association with SU. The mean was 0.38 (SD 0.14) in 2019 and saw a small increase to mean 0.39 (SD 0.13) in 2020, with a negligible effect size (d=0.07). This slightly elevated association remained constant in 2021, with a mean of 0.39 (SD 0.13). A notable trend was observed for neuroticism. Its association with SU was a mean of 0.63 (SD 0.15) in 2019. Contrary to what might be expected under pandemic-related stress, this association slightly decreased to 0.61 (SD 0.15) in 2020, a change with a small negative effect size (d=−0.13). The association then remained stable at this level in 2021. Finally, the association between extraversion and SU exhibited considerable stability. The mean association was 0.53 (SD 0.14) in 2019 and remained unchanged in both 2020, mean 0.53 (SD 0.14) and 2021, mean 0.53 (SD 0.14), with the analysis indicating no significant change (*P≥*.99). In summary, the results indicate that while the association of extraversion with SU remained constant, openness, conscientiousness, agreeableness, and neuroticism showed varying degrees of change in their associations with SU between 2019 and 2021, with the most pronounced shifts occurring in 2020.

### Personality Traits as Predictors of SU

**Figure 2.**
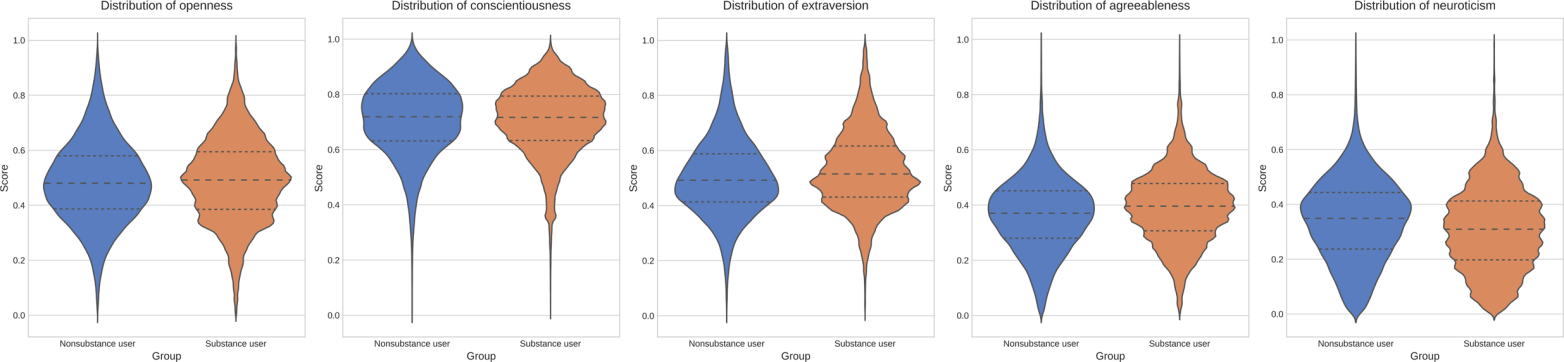
Distribution of the Big Five personality trait scores for nonsubstance user and substance user groups.

To investigate the relationship between personality and SU, we first examined the distributional differences in the Big Five personality scores between the SU and nonsubstance user groups on a sample corpus of 133,374. As illustrated in Figure 2, a visual inspection of the data reveals distinct patterns. The distributions for extraversion and agreeableness are visibly shifted higher for the SU group, while the distribution for neuroticism is shifted lower. In contrast, the distributions for openness and conscientiousness appear largely similar between the 2 groups.

**Figure 3.**
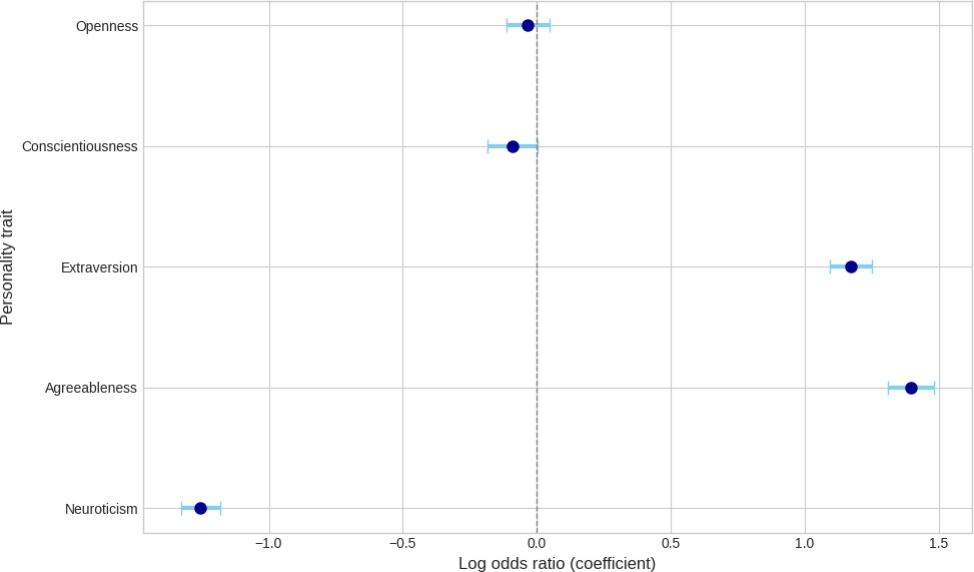
Log odds ratios of personality traits on substance use.

To formally test these observed differences and determine the unique contribution of each trait, we conducted a multiple logistic regression analysis on a balanced sample of 133,374 corpus (n=66,687 per group). The analysis used the dichotomous SU classification as the dependent variable, with the 5 continuous personality scores as independent variables. The overall model was statistically significant, indicating that personality traits reliably distinguished between the 2 groups. The OR and 95% CIs from the model are presented in Figure 3.

The analysis identified 3 statistically significant predictors. Agreeableness demonstrated a strong positive association with SU (OR 4.04, 95% CI 3.71-4.41, *P*<.001). Extraversion also emerged as a robust positive predictor (OR 3.22, 95% CI 2.98-3.49, *P*<.001). In contrast, a significant negative relationship was observed for neuroticism (OR 0.29, 95% CI 0.26-0.31, *P*<.001).

Neither conscientiousness (OR 0.91, 95% CI 0.83-1.00, *P*=.055) nor openness (OR 0.97, 95% CI 0.89-1.05, *P*=.42) was found to be a statistically significant predictor in the final model, although the association for conscientiousness approached statistical significance.

### Emotion Association in Personality Traits

**Figure 4.**
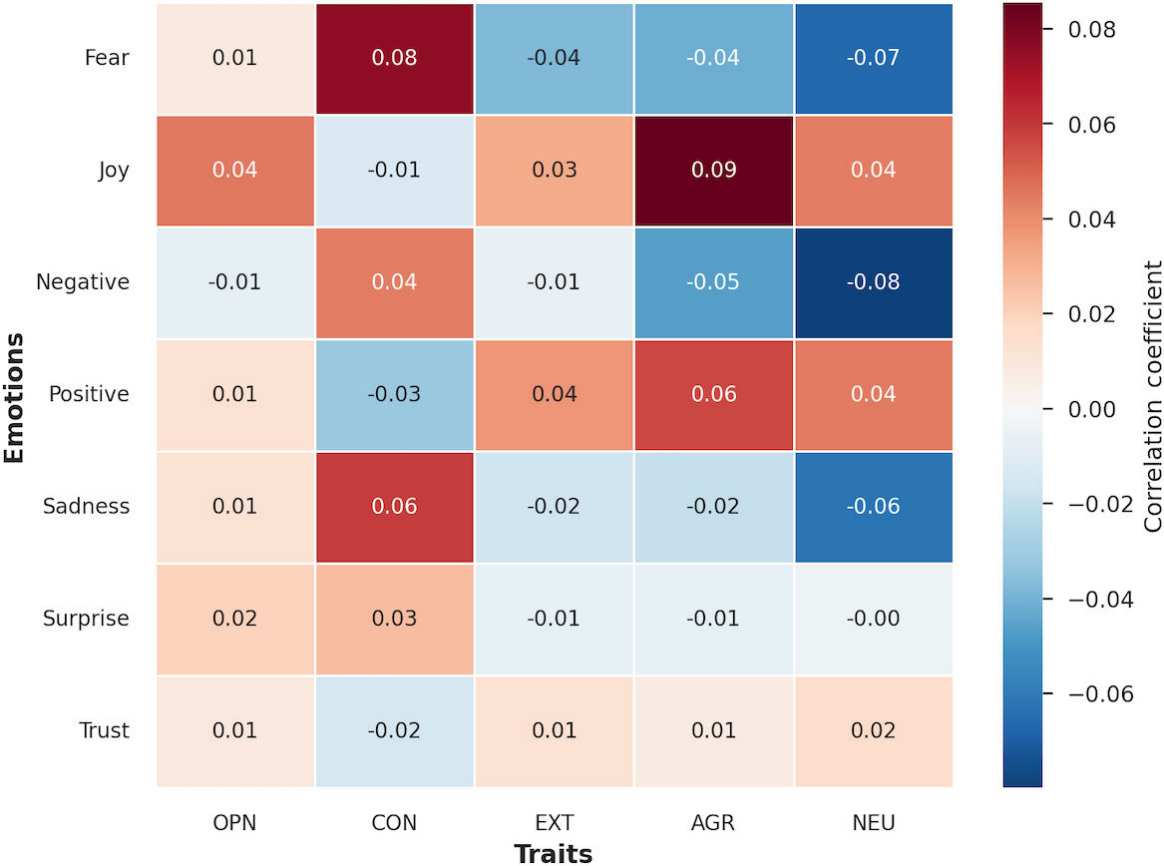
Heatmap correlation between personality traits and emotion markers. AGR: agreeableness; CON: conscientiousness; EXT: extraversion; NEU: neuroticism; OPN: openness.

To explore the relationship between personality and emotional expression, a biserial correlation analysis was performed on a 2020 corpus of SU-related online posts. The analysis compared 5 personality traits against 7 emotion categories: namely, fear, joy, negative, positive, sadness, surprise, and trust, yielding generally weak to modest correlations, with absolute coefficient values ranging from 0.00 to 0.09, as detailed in Figure 4.

The findings indicated distinct emotional patterns for certain traits. Agreeableness showed the strongest positive association with positive emotionality, correlating most highly with joy (*r*=.09) and positive emotions (*r*=.06). In contrast, conscientiousness was linked to negative valence emotions, showing positive correlations with fear (*r*=.08) and sadness (*r*=.06). A counterintuitive pattern was observed for neuroticism, which was negatively correlated with the expression of negative emotions (*r*=−.08), fear (*r*=−.07), and sadness (*r*=−.06). Correlations for extraversion and openness were weaker and less distinct across all emotion categories.

### Personality Traits as Predictors of Various Substance Types

Multilevel logistic regression analyses were conducted to investigate the association between 5 core personality traits (openness, conscientiousness, extraversion, agreeableness, and neuroticism) and the likelihood of using various types of substances in the 2020 corpus. The findings for each personality trait are detailed below, referencing the estimated log ORs (coefficients) and their 95% CIs.

### Openness

**Figure 5.**
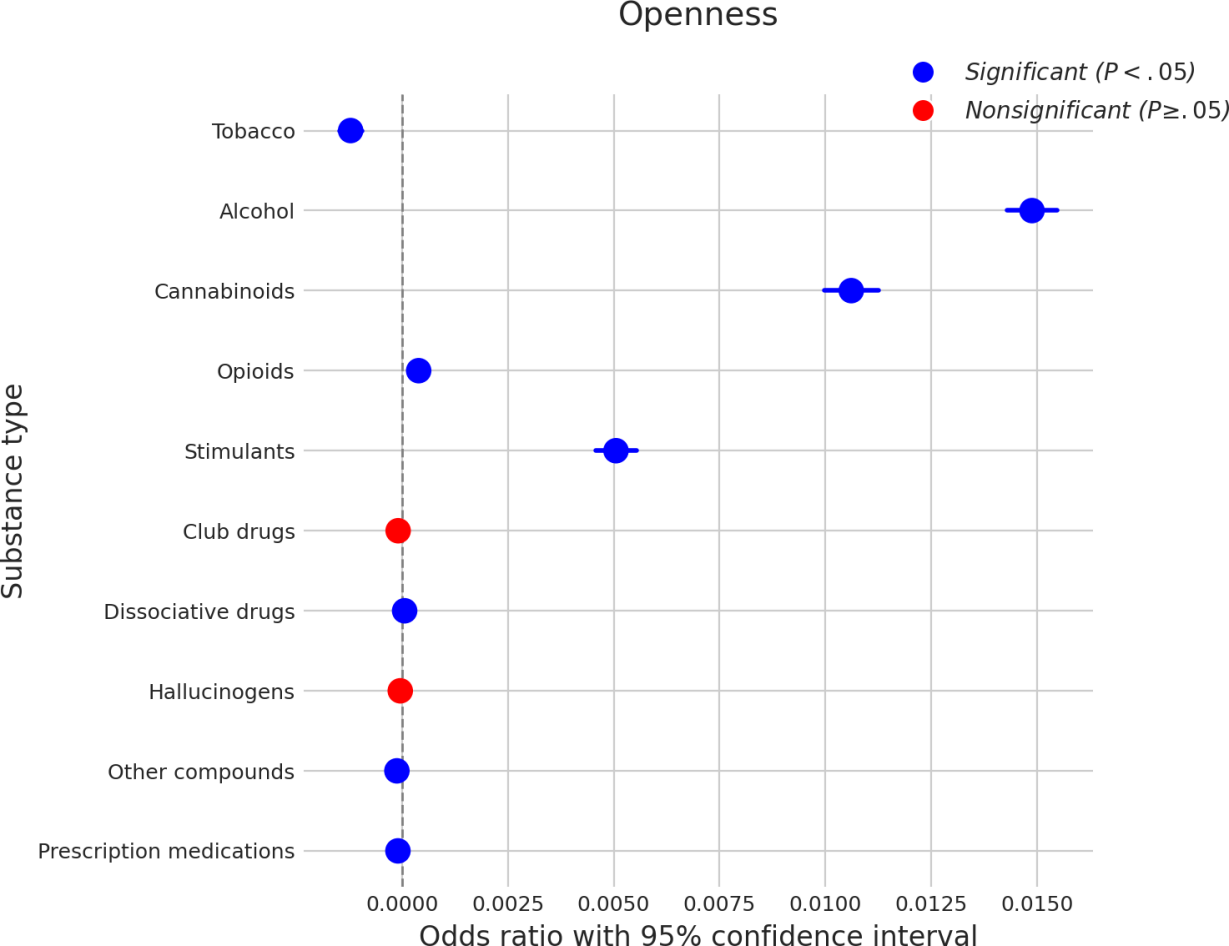
Log odds ratios (and 95% CIs) for the association between openness and various substance types.

As shown in Figure 5, openness demonstrated several significant associations with SU. Higher levels of openness were significantly associated with an increased likelihood of using alcohol, with a coefficient of approximately 0.014, and cannabinoids, with a coefficient of approximately 0.010. A similar positive association was found with stimulants, yielding a coefficient of approximately 0.005. Furthermore, openness was significantly associated with an increased likelihood of tobacco use (coefficient≈0.0005), opioid use (coefficient≈0.001), dissociative drug use (coefficient≈0.001), the use of other compounds (coefficient≈0.001), and the use of prescription medications (coefficient≈0.0005). For all these latter substances, the effect sizes indicated a slightly increased likelihood. In contrast, nonsignificant associations were observed between openness and the use of club drugs and hallucinogens.

### Conscientiousness

**Figure 6.**
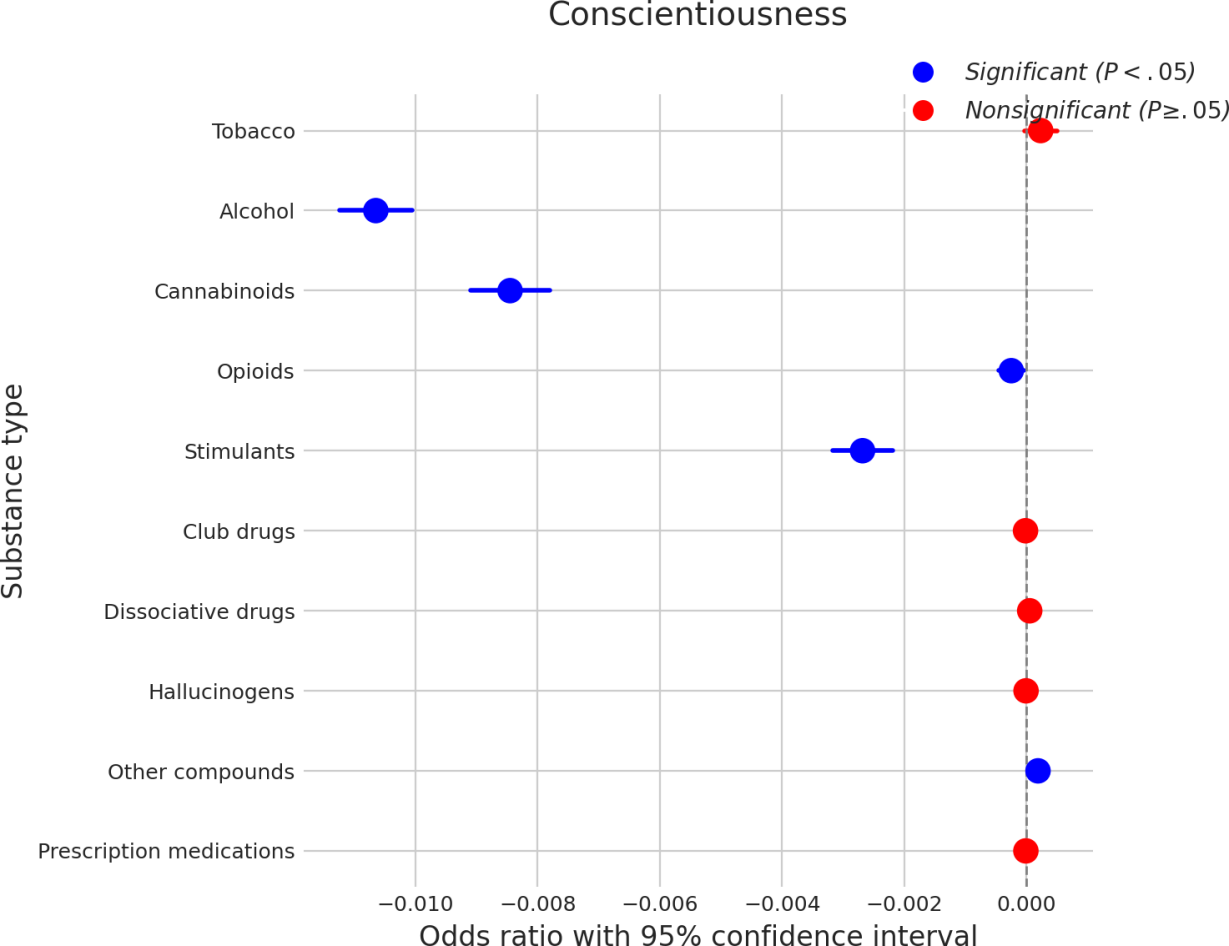
Log odds ratios (and 95% CIs) for the association between conscientiousness and various substance types.

Figure 6 illustrates the relationship between conscientiousness and SU. Higher conscientiousness was significantly associated with a decreased likelihood of using alcohol, indicated by a coefficient of approximately −0.011. Similarly, increased conscientiousness was linked to a reduced probability of using cannabinoids (coefficient≈−0.008) and stimulants (coefficient≈−0.003). Conversely, a somewhat counterintuitive finding was that higher conscientiousness was significantly associated with a slightly increased likelihood of using opioids (coefficient≈0.001) and other compounds (coefficient≈0.0005). There were no significant associations found between conscientiousness and the use of tobacco, club drugs, dissociative drugs, hallucinogens, or prescription medications.

### Extraversion

**Figure 7.**
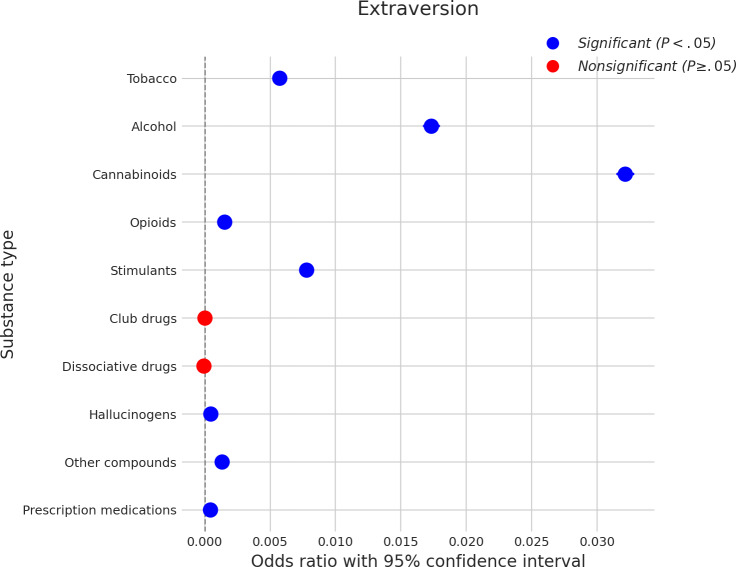
Log odds ratios (and 95% CIs) for the association between extraversion and various substance types.

Extraversion showed widespread significant positive associations with SU, as depicted in Figure 7. A higher degree of extraversion was significantly associated with an increased likelihood of using cannabinoids, which showed the largest effect size for this trait (coefficient≈0.031). Positive associations were also found for alcohol (coefficient≈0.017), stimulants (coefficient≈0.009), and tobacco (coefficient≈0.006). Additionally, higher extraversion was linked to an increased likelihood of using opioids (coefficient≈0.002), hallucinogens (coefficient≈0.002), other compounds (coefficient≈0.002), and prescription medications (coefficient≈0.001). Nonsignificant associations were observed between extraversion and the use of club drugs and dissociative drugs.

### Agreeableness

**Figure 8.**
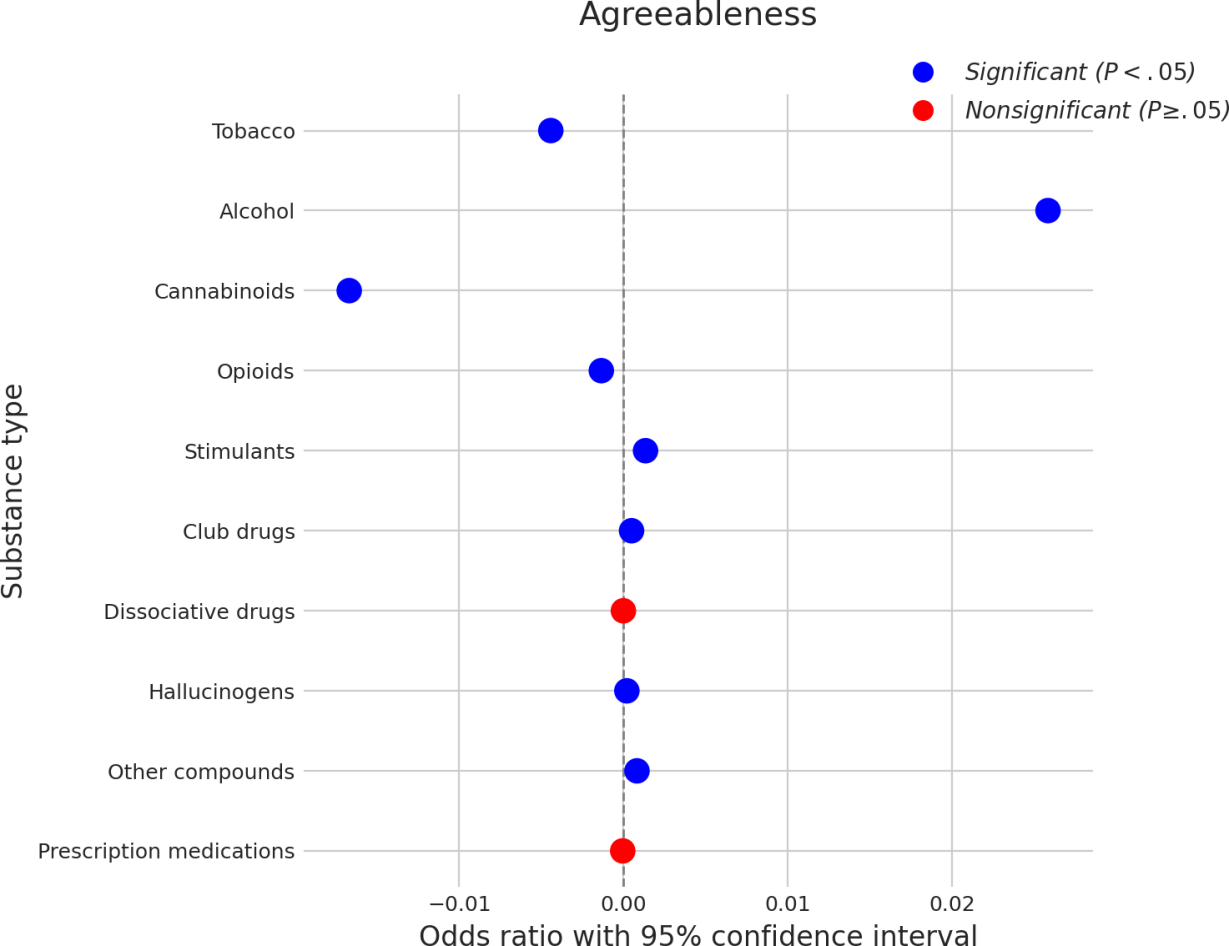
Log odds ratios (and 95% CIs) for the association between agreeableness and various substance types.

The associations between agreeableness and SU are presented in Figure 8. Higher agreeableness was significantly associated with a decreased likelihood of using cannabinoids, with a coefficient of approximately −0.012, and tobacco, with a coefficient of approximately −0.008. Intriguingly, higher agreeableness was also significantly associated with an increased likelihood of using several substances. The most notable positive association was with alcohol (coefficient≈0.023). Furthermore, increased agreeableness was linked to a higher probability of using opioids (coefficient≈0.003), stimulants (coefficient≈0.002), club drugs (coefficient≈0.001), hallucinogens (coefficient≈0.001), and other compounds (coefficient≈0.001). No significant associations were found between agreeableness and the use of dissociative drugs or prescription medications.

### Neuroticism

**Figure 9.**
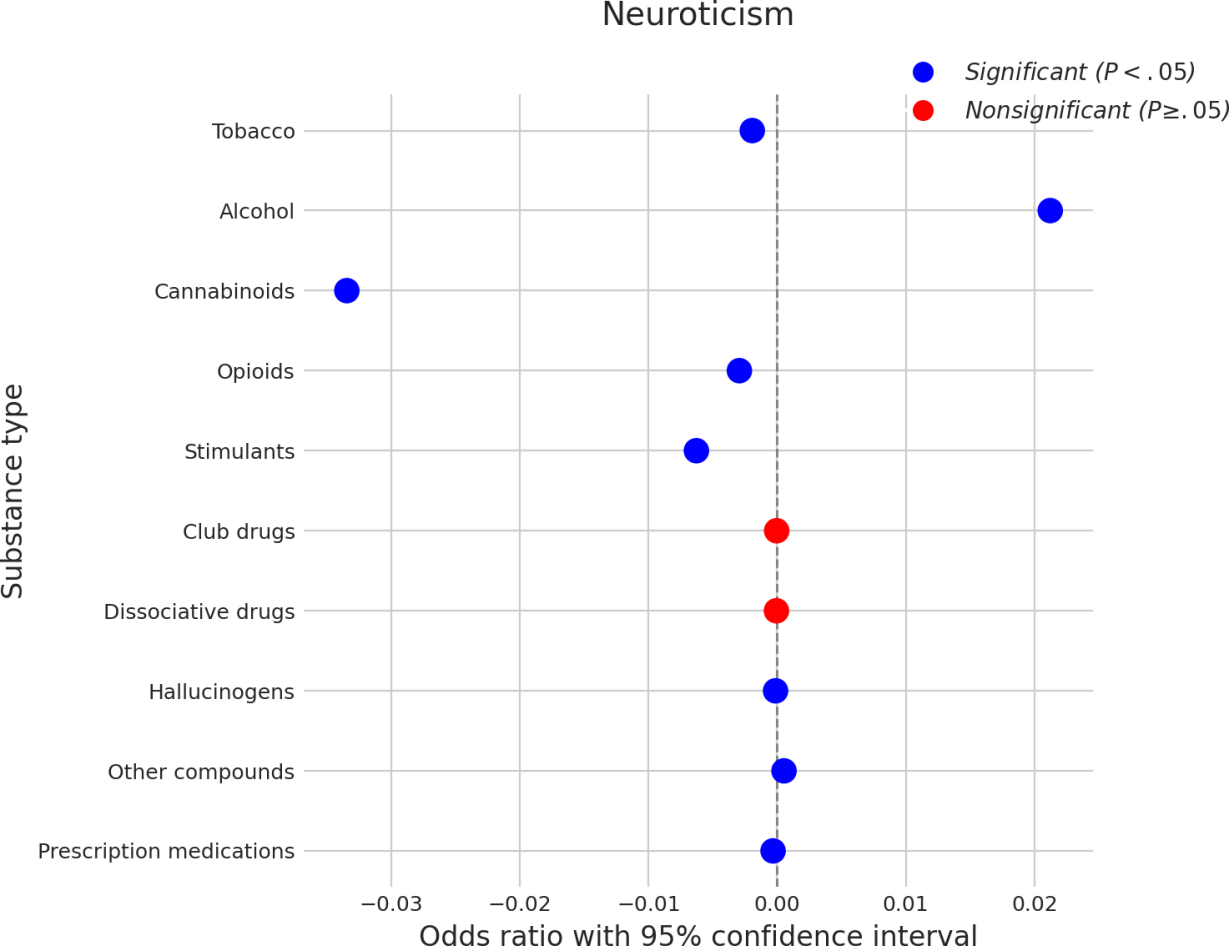
Log odds ratios (and 95% CIs) for the association between neuroticism and various substance types.

Figure 9 displays the results for neuroticism. Higher neuroticism was significantly associated with an increased likelihood of using alcohol, with a coefficient of approximately 0.020. Positive associations, though smaller in magnitude, were also observed for hallucinogens (coefficient≈0.001) and other compounds (coefficient≈0.001). A very small but statistically significant positive association was also noted with prescription medications (coefficient near 0). Conversely, higher neuroticism was significantly associated with a decreased likelihood of using several other substances. The strongest negative association was with cannabinoids (coefficient≈−0.030). Other significant negative associations were found for stimulants (coefficient≈−0.007), tobacco (coefficient≈−0.005), and opioids (coefficient≈−0.004). Nonsignificant associations were found between neuroticism and the use of club drugs and dissociative drugs.

### Personality Traits Association With Age and Gender

This section details the findings from a multilevel logistic analysis examining the interaction between personality traits and demographic factors (age group and sex) in relation to SU. The results are presented as ORs with 95% CIs. An OR greater than 1.00 indicates that a personality trait’s association with SU is stronger (or less protective) in the index group (teenagers or females) compared to the reference group (adults or males). Conversely, an OR less than 1.00 suggests the trait’s association is weaker (or more protective) in the index group. All reported effects are statistically significant, as indicated by CIs not crossing 1.00, owing in part to the large sample sizes.

To better capture demographic-specific patterns and potential nonlinear effects of age and sex on personality-SU associations, we stratified the analyses by age group and gender rather than including these variables solely as covariates. This stratification enables a clearer depiction of how personality traits influence SU discourse within distinct subgroups, highlighting developmental and sex-specific variations that might be obscured in aggregate models.

### Personality Traits and SU: Teenagers Versus Adults

**Figure 10.**
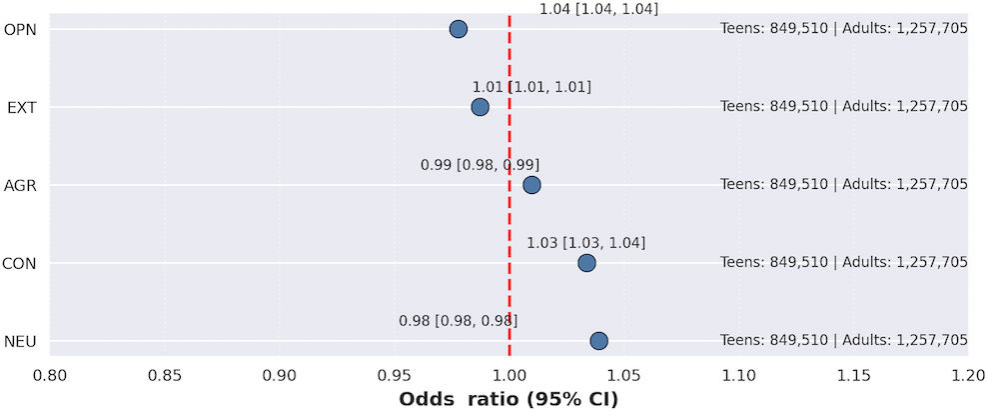
Personality traits associated with teenagers’ substance use versus adults’ substance use. AGR: agreeableness; CON: conscientiousness; EXT: extraversion; NEU: neuroticism; OPN: openness.

Figure 10 illustrates the differential association of personality traits with SU in teenagers (n=849,510) compared to adults (n=1,257,705). The association between openness and SU was found to be significantly stronger among teenagers than adults (OR 1.04, 95% CI 1.04-1.04). Similarly, extraversion’s association with SU was slightly stronger in teenagers compared to adults (OR 1.01, 95% CI 1.01-1.01). In contrast, the association of agreeableness with SU, which is typically protective, was found to be slightly more pronounced (more protective) in teenagers relative to adults (OR 0.99, 95% CI 0.98-0.99). Conscientiousness, generally a protective trait, exhibited a significantly weaker protective association with SU in teenagers when compared to adults (OR 1.03, 95% CI 1.03-1.04), suggesting that the protective effect of conscientiousness against SU is attenuated in adolescence. Finally, the association of neuroticism with SU, typically a risk factor, was found to be slightly weaker in teenagers compared to adults (OR 0.98, 95% CI 0.98-0.98).

### Personality Traits and SU: Females Versus Males

**Figure 11.**
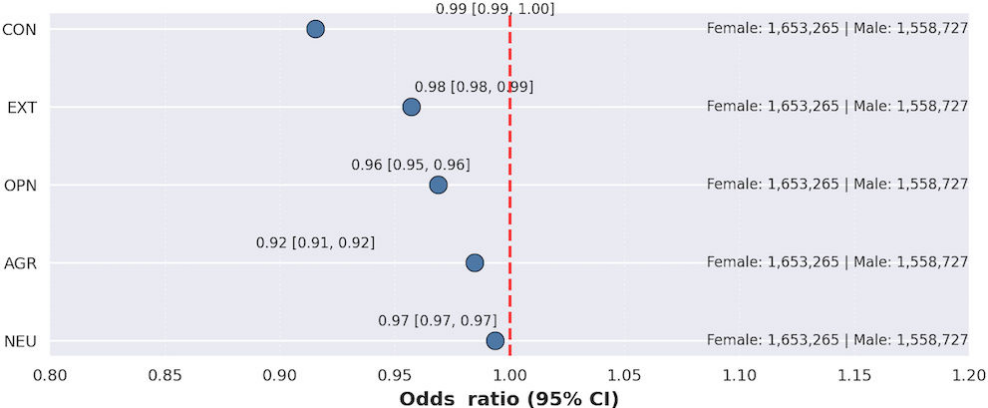
Personality traits associated with male substance use versus female substance use. AGR: agreeableness; CON: conscientiousness; EXT: extraversion; NEU: neuroticism; OPN: openness.

Figure 11 presents the differential association of personality traits with SU in females (n=1,653,265) compared to males (n=1,558,727). The protective association of conscientiousness with SU was slightly stronger in females than in males (OR 0.99, 95% CI 0.99-1.00). Extraversion’s association with SU was weaker among females compared to males (OR 0.98, 95% CI 0.98-0.99), and the association of openness with SU was also weaker in females relative to males (OR 0.96, 95% CI 0.95-0.96). Agreeableness demonstrated a substantially stronger protective association with SU in females compared to males (OR 0.92, 95% CI 0.91-0.92). Lastly, neuroticism’s association as a risk factor for SU was weaker in females compared to males (OR 0.97, 95% CI 0.97-0.97).

### Topic Analysis

This section presents key themes or topics discussed within each personality trait in SU discourse. We conducted the BERTopic analysis to identify thematic clusters within a corpus of documents from 2020 related to personality traits and SU. The analysis was performed separately for documents associated with each of the Big Five personality traits. For each trait, the most prominent topics, their corresponding document counts, and their proportion relative to the total documents for that trait are reported in Table 2.

**Table 2.** BERTopic analysis result.

Topic	OpenAI label	Document, n (%)
Openness		
0	Openness to nightlife enjoyment	134,265 (54.42)
1	Openness to addiction	92,386 (37.45)
2	Political negligence	8246 (3.34)
3	Public health opinions	3100 (1.26)
4	Change in supply and distribution	2898 (1.17)
5	Innovative service improvements	2626 (1.06)
6	Political retrograde	1963 (0.8)
7	Stress drinking	1077 (0.44)
8	Embracing normalization	74 (0.3)
9	Embracing fully	62 (0.3)
Conscientiousness		
0	Responsible drinking habits	149,860 (52.78)
1	Responsible smoking habits	124,349 (43.80)
2	Political retrograde and study	3641 (1.28)
3	Homeless advocacy efforts	1398 (0.49)
4	Contract liquidation assessment	1316 (0.46)
5	Consistent technology upgrades	1103 (0.39)
6	Climate change impact analysis	969 (0.34)
7	Maine senator Susan Collins	850 (0.3)
8	Religious attributes	353 (0.12)
9	Organized and responsible mindset	91 (0.03)
Extraversion		
0	Social drinking behavior	150,361 (48.46)
1	Social smoking habits	140,837 (45.40)
2	Political events	13,560 (4.37)
3	School discussion	2571 (0.83)
4	Cancellation and bankruptcy	1143 (0.37)
5	Normalize the change	1064 (0.34)
6	Discussion about deaths	303 (0.1)
7	Energetic socialization behavior	232 (0.07)
8	Normalize social interaction	118 (0.04)
9	Outgoing enthusiast	56 (0.02)
Agreeableness		
0	Friendly beverage choices	136,175 (54.48)
1	Social drinking habits	99,238 (39.70)
2	Nobody protests peacefully, agitated	6852 (2.74)
3	Changes in supply and distribution	2894 (1.16)
4	Employee and contracts	2278 (0.91)
5	School and eligible discussion	1191 (0.48)
6	Normalize the change	558 (0.22)
7	Weather demands	442 (0.18)
8	Gentle pain relief	292 (0.12)
9	Harmony discussion	53 (0.02)
Neuroticism		
0	Emotionally turbulent drinking habits	123,209 (50.05)
1	Drink and anxiety association	95,229 (38.69)
2	Anxiety during the COVID pandemic	10,570 (4.29)
3	Contract liquidation and upgrade	8186 (3.33)
4	Political tension	7036 (2.86)
5	Anxious hopes	854 (0.35)
6	Normalize the change	565 (0.23)
7	Medications	227 (0.09)
8	Liquid and drinks	174 (0.07)
9	Cross-border issues	105 (0.04)

A total of 246,700 documents were analyzed in relation to openness. The 2 most dominant topics identified were “openness to nightlife enjoyment” (topic 0) accounting for 54.42% (n=134,265) of the documents, and “openness to addiction” (topic 1) comprising 37.45% (n=92,386). A smaller, yet notable topic, “stress drinking” (topic 7), appeared in 0.44% (n=1077) of documents. Other topics, such as “political negligence” (n=8246, 3.34%), “public health opinions” (n=3100, 1.26%), and “change in supply and distributions” (n=2898, 1.17%), represented smaller proportions and appeared less directly related to SU behaviors per se, potentially reflecting broader contextual discussions within the dataset.

For conscientiousness, 283,930 documents were analyzed. The analysis revealed 2 primary topics: “responsible drinking habits” (topic 0) which constituted 52.78% (n=149,860) of the documents, and “responsible smoking habits” (topic 1) accounting for 43.80% (n=124,349) of them. A very small proportion was attributed to “organized and responsible mindset” (topic 9) at 0.03% (n=91). The remaining topics, such as “political retrograde and study” (n=3641, 1.28%) and “homeless advocacy efforts” (n=1398, 0.49%) were less prevalent and less clearly connected to individual SU patterns.

The analysis of 310,245 documents related to extraversion highlighted “social drinking behavior” (topic 0) as the most significant topic, with 48.46% (n=150,361) of the documents. Closely following was “social smoking habits” (topic 1) representing 45.40% (n=140,837). Smaller topics included “energetic socialization behavior” (topic 7) at 0.07% (n=232) and “outgoing enthusiast” (topic 9) at 0.02% (n=56). Topics such as “political events” (n=13,560, 4.37%) and “school discussion” (n=2571, 0.83%) were also present but had a less direct bearing on SU itself.

A total of 249,973 documents were analyzed for agreeableness. The most prominent topic was “friendly beverage choices” (topic 0) accounting for 54.48% (n=136,175) of documents. The second major topic was “social drinking habits” (topic 1) comprising 39.70% (n=99,238). A topic labeled “gentle pain reliefs” (topic 8) was found in 0.12% (n=292) of documents. Other identified topics, such as “nobody protests peacefully, agitated” (n=6852, 2.74%) and “changes in supply and distributions” (n=2894, 1.16%) were less frequent, and their connection to agreeableness in the context of SU was not immediately apparent from the labels.

For neuroticism, 246,155 documents were processed. The leading topic was “emotionally turbulent drinking habits” (topic 0) representing 50.05% (n=123,209) of the documents. The second significant topic was “drink and anxiety association” (topic 1) with 38.69% (n=95,229). Another relevant topic, “anxiety during COVID pandemic” (topic 2) accounted for 4.29% (n=10,570). The topic “medications” (topic 7) appeared in 0.09% (n=227) of documents.

## Discussion

### Principal Findings

The principal finding of this research underscores the paramount importance of social-relational personality traits in predicting SU within the studied online communities.

Our analysis identifies extraversion (OR 3.22, 95% CI 2.98-3.49) and, most powerfully, agreeableness (OR 4.04, 95% CI 3.71-4.41) as the strongest positive predictors of SU. This suggests that, within this digital sphere, SU discourse is deeply embedded in social and affiliative contexts, a conclusion reinforced by topic analyses revealing prominent themes of “social drinking behavior” and “friendly beverage choices.”

In stark contrast to this socially driven pattern, and challenging long-held theoretical assumptions, our study reveals a paradoxical and robust protective effect of neuroticism. This counterintuitive finding is consistently supported across our analyses: neuroticism was strongly and negatively associated with SU classification (OR 0.29, 95% CI 0.26-0.31), its link to SU discussions diminished during the 2020 pandemic peak (d=−0.13), and it was negatively correlated with the online expression of negative emotions (r=−.08). This result directly opposes the traditional self-medication hypothesis, which posits that individuals use substances to cope with negative affect [43]. A potential explanation lies in online expression; individuals high in neuroticism may be less inclined to participate in public substance-related discussions due to concerns over social judgment or reputational risk, even if they engage in SU offline. Moreover, negative emotions might be managed through private coping mechanisms rather than overt online disclosure, while SU-related conversations appear to attract users with social-affiliative traits, such as extraversion and agreeableness. Together, these patterns indicate that online SU discourse reflects the social and performative functions of communication, highlighting a distinction between public engagement and direct behavioral risk, and providing a nuanced understanding of personality influences in digital contexts.

Collectively, these findings suggest that in contemporary online environments, the drivers of SU discourse may pivot away from solitary emotional coping and toward functions of social connection. While the pandemic provided a salient natural context that accentuated these tendencies, it is equally plausible that these patterns reflect broader digital-era dynamics of social affiliation and positive self-expression, consistent with emerging perspectives in online health behavior [29] and positive psychology.

### RQ1: Longitudinal Variation: Did the Linguistic Expression of Big Five Personality Traits in SU-Related Discourse on Twitter Exhibit Significant Longitudinal Variation Between 2019-2021, and What Was the Measurable Impact of the COVID-19 Pandemic on These Temporal Patterns?

Our longitudinal analysis confirms the COVID-19 pandemic acted as a significant catalyst, altering the linguistic expression of personality within SU discourse on Twitter. The year 2020 saw an increase in language related to openness, likely reflecting an exploration of novel coping mechanisms during a period of uncertainty [44], and conscientiousness. The latter’s increase does not suggest more SU, but rather a heightened discourse around regulation and control as conscientious individuals grappled with disrupted routines and health anxieties [45].

Most critically, the linguistic markers for neuroticism significantly decreased, directly challenging the self-medication hypothesis [43], which would predict a surge. This counterintuitive finding may be explained by “affective blunting,” where substances effectively numb distress and thus reduce its linguistic expression, or by a “discourse displacement,” where universal pandemic anxiety became a less specific theme to tie to SU. Meanwhile, the stability of extraversion underscores that the core function of SU discourse as a medium for social connection remained robust despite physical distancing. These findings illustrate that the pandemic did not simply amplify existing trends but fundamentally reshaped the content of online SU conversations.

### RQ2: Predictive Analysis: To What Extent Do the Big Five Personality Traits Predict SU Classification Among Individuals on Platform Twitter During the Unique Socioenvironmental Context of the COVID-19 Pandemic?

Our predictive analysis reveals a personality profile of online SU discourse that fundamentally prioritizes social-relational functions over individual distress, presenting a direct challenge to established risk models. The 2 strongest predictors for being classified as an SU were agreeableness (OR 4.04, 95% CI 3.71-4.41) and extraversion (OR 3.22, 95% CI 2.98-3.49). While the link to extraversion aligns with theories of sensation-seeking and sociability [46], the powerful, positive association with agreeableness is highly novel. It starkly contrasts with a large body of literature that typically links low agreeableness to SUDs [6]. This suggests that on a public platform such as Twitter, SU discourse is not an act of antagonism or noncompliance but rather a prosocial behavior, where agreeable individuals engage in communal sharing and social bonding around the topic.

Perhaps most consequentially, neuroticism emerged as a robust protective factor (OR 0.29, 95% CI 0.26-0.31). This finding directly contradicts the canonical self-medication hypothesis, which posits that individuals high in neuroticism are more likely to use substances to cope with negative affects [43]. Our results indicate that on this platform, individuals exhibiting higher neuroticism are significantly less likely to be part of the SU discourse community. This does not necessarily mean they abstain from SU, but rather that their potential distress does not translate into public, online identification with such behaviors. They may use other coping mechanisms or simply separate their internal emotional state from this specific form of social performance.

The nonsignificance of openness and conscientiousness in this predictive model suggests their primary role is not in determining if one joins the SU conversation, but rather in shaping the content and framing of that conversation once engaged. Ultimately, our findings make a critical distinction: personality on platform Twitter appears to predict engagement in the socially mediated discourse of SU, a behavior that is functionally distinct from the private act of consumption itself.

### RQ3: Emotional Expression: How Did the Emotional Tone of SU-Related Discourse Vary Across Individuals With Different Dominant Personality Traits Throughout the Pandemic?

Examining emotional expression during the 2020 pandemic peak reveals how personality shapes the affective tone of SU discourse. The link between conscientiousness and emotions of fear and sadness points to a form of “responsible anxiety,” where discourse was imbued with legitimate concerns about health and control amidst a crisis [45]. In contrast, the association of agreeableness with joy and positivity highlights its role as a “social glue,” with individuals likely framing SU in a positive, communal light to foster support and morale during a period of isolation.

The most telling result is the negative correlation between neuroticism and the expression of negative emotions. This provides strong support for an “affective blunting” or suppression hypothesis, suggesting that for these individuals, SU discourse reflects a state where negative feelings have been dampened, rather than a platform for cathartic expression. This challenges us to consider that for some, SU discussions may signify emotional regulation and suppression, not just the articulation of distress.

### RQ4: Substance Specificity: What Were the Associations Between Inferred Personality Traits and Discourse Related to Specific Substance Types?

Disaggregating SU by substance type reveals critical, personality-driven patterns. The association between neuroticism and alcohol discourse supports a specified self-medication model, where individuals turned to a legal and accessible anxiolytic to cope with pandemic-induced distress [47]. This finding provides a personality-based lens on the widely reported increase in alcohol consumption during lockdowns.

Conversely, openness to experience was linked to discussions of cannabinoids and hallucinogens, consistent with the trait’s connection to novelty-seeking [5]. This discourse likely reflects a form of “cognitive escapism” from the monotony of confinement. Finally, extraversion remained tied to socially contextualized substances such as alcohol and cannabis, with discourse focused on adapting social rituals to a virtual environment. These findings confirm that motivations for use are codetermined by personality and substance, necessitating targeted, rather than generic, public health messaging.

### RQ5: Demographic Dimensions: How Did the Relationships Between Personality and SU Discourse Differ Across Key Demographic Factors, Specifically Age and Gender?

Our findings confirm that age and sex are crucial moderators of personality’s influence on SU discourse. In adolescence, the observed patterns, such as a diminished protective effect of conscientiousness and amplified influence of extraversion, align with developmental neuroscience models of a maturing prefrontal cortex and a hypersensitive social reward system. This highlights a unique developmental window of personality-based risk and resilience.

Furthermore, we identified significant sex differences. The stronger protective effects of agreeableness and conscientiousness in females align with established gender differences in personality and risk-aversion [24]. Critically, risk-associated traits such as neuroticism and extraversion had weaker associations with SU discourse in females, suggesting different, possibly more internalizing or relational, pathways to SU. These results underscore the necessity of moving beyond monolithic models of risk to develop intersectional, demographic-specific prevention strategies.

### RQ6: Thematic Content: What Were the Prevailing Topics Within SU Discourse, and How Did These Themes Differ Across the Five Major Personality Traits During the Pandemic?

#### Overview

The topic analysis revealed distinct “narrative frameworks” for SU that are patterned by personality, providing a clear basis for tailored public health interventions. The discourse of neuroticism centered on “emotional coping,” suggesting that messaging for this group should focus on alternative emotional regulation strategies [48]. In contrast, the extraversion and agreeableness narratives focused on “social facilitation,” indicating a need for interventions that target harm reduction in social settings and promote nonsubstance-based social activities.

The dual themes of “sensation-seeking” and “addiction awareness” for openness highlight a user base receptive to sophisticated, fact-based harm reduction information. Finally, the conscientiousness discourse on “controlled behavior” suggests this group would be responsive to tools for self-monitoring and goal-setting. By identifying these personality-specific discursive fingerprints, we can move beyond one-size-fits-all campaigns to develop more resonant and effective public health communications in the digital age.

#### Theoretical and Practical Implications

The implications of this social-centric model are significant for both theory and practice. Our findings demand that researchers distinguish between the private act of substance consumption and the mention of the usage of substances in online discourse. This provides a direct blueprint for personality-tailored public health interventions: focusing on harm reduction in social settings for individuals high in agreeableness and extraversion, while offering alternative coping strategies for the “emotional coping” narratives associated with neuroticism [48]. Acknowledging the present study’s limitations, future work should validate these findings across different platforms and integrate them with offline clinical data to build a more holistic and effective framework for addressing SU risk in the digital age.

#### Policy Implications

From a policy perspective, these results underscore the importance of targeting the social drivers of SU discourse online. Prevention strategies should go beyond individual-level risk and address the communal contexts where SU is normalized, particularly among highly agreeable and extraverted users. The protective effect of neuroticism highlights the need for adaptive, data-driven monitoring that can refine early-warning systems during public health crises. Policy makers could leverage NLP-based tools for real-time trend detection while partnering with technology companies to embed harm-reduction interventions, such as in-platform nudges or targeted public service announcements. To ensure equity, these strategies must be accompanied by safeguards that protect user privacy, mitigate stigmatization, and promote algorithmic transparency. By aligning digital health policies with personality-informed insights, governments and health agencies can better anticipate, monitor, and reduce SU-related harms in the digital era.

### Limitations

The findings of this study, while offering valuable insights into the dynamics of SU discourse on social media, must be interpreted in light of several important limitations.

A primary constraint stems from our reliance on probabilistic inference models to ascertain user attributes. Although our classifiers achieved approximately 80% accuracy, inferred data are proxies, not ground-truth information, and the potential for misclassification can introduce bias that propagates through the analysis. This is compounded by our use of an archival dataset from Twitter [31], where data inconsistencies could impact the precision of longitudinal analyses. Furthermore, this study’s scope is confined to English-language discourse on Twitter, which limits the generalizability of our findings to other platforms, cultures, or languages where SU expression differs.

An important conceptual limitation concerns the treatment of engagement in SU discourse as a proxy for actual SU behavior. Users may participate in substance-related discussions for social, performative, or cultural reasons without personally consuming substances. Consequently, the presence of SU-related posts should not be interpreted as definitive evidence of individual SU. While this proxy enables large-scale, population-level analyses, it constrains the ability to draw causal inferences about personal behavior and necessitates caution in interpreting associations between personality traits and SU engagement online.

Another limitation lies in the application of a personality model trained on Reddit data to Twitter users. While prior research has shown that language-based personality inference models can generalize reasonably well across platforms [49], differences in user demographics, community norms, and writing styles between Reddit and Twitter may introduce systematic biases. For example, Reddit’s longer-form discussions differ from the brevity and immediacy of tweets, which could influence the linguistic markers of personality traits. Thus, while the use of a well-validated model enables scalable personality assessment, its cross-platform application must be interpreted cautiously.

Finally, our demographic analysis is constrained by the nonrepresentative user base of Twitter, which is known to skew toward the younger and more urban. In addition, demographic inference models themselves may introduce biases. For example, gender classifiers are limited in recognizing nonbinary or gender-diverse individuals, and other attributes such as race or age may be misclassified due to sparse or ambiguous linguistic signals. Without reliable platform-wide demographic benchmarks, it is impossible to fully normalize our findings, meaning observed differences between groups could reflect both representation disparities and limitations of demographic inference rather than true behavioral patterns.

Additionally, while this study spans 2019‐2021, our focus is on understanding personality-SU relationships in online discourse, treating the pandemic as a contextual societal shock. Descriptive data from 2019 and 2021 provide a reference point, but this study does not perform formal comparisons between crisis and normal conditions; such analyses are considered a direction for future research.

### Future Work

Building on the findings and limitations of this study, several key avenues for future research emerge. These directions focus on enhancing methodological rigor, expanding the scope of analysis for greater generalizability, and integrating diverse data sources for a more holistic understanding.

First, future work should prioritize the refinement and validation of the computational models used for inference. A critical next step is to conduct rigorous domain adaptation testing to evaluate and improve the performance of models trained on one platform (Reddit) and applied to another (Twitter), as was the case for our personality classifier. This would help account for platform-specific linguistic norms and reduce potential bias. Similarly, developing more transparent and inclusive demographic inference models, trained on more diverse and representative datasets, is essential for improving the accuracy and equity of these computational tools.

Second, the scope of the research should be significantly broadened to enhance the generalizability of the findings. This involves expanding the analysis to include multiple social media platforms (such as Reddit, 2024 [50], TikTok, 2024 [ByteDance] [51], and Facebook, 2024 [Meta] [52]) to capture a more complete picture of the digital ecosystem. Concurrently, future studies must move beyond an English-language focus by leveraging multilingual NLP models. Such an endeavor would not only provide a more global perspective on SU discourse but would also necessitate interdisciplinary collaborations with social scientists, ethicists, and researchers fluent in other languages to ensure the culturally competent application of these methods.

Finally, to overcome the inherent limitations of using social media data alone, future research should aim to triangulate computational findings with traditional offline data sources. Integrating large-scale social media analyses with clinical records, ecological momentary assessments, or targeted surveys would allow researchers to validate digital proxies against ground-truth behaviors and clinical outcomes. This mixed-methods approach would provide a much richer, multilayered understanding of the complex interplay between personality, SU, and mental health, ultimately strengthening the foundation for developing more effective and responsive public health interventions.

### Conclusions

This research fundamentally reconfigures the understanding of SU in digital spaces, revealing that online discourse is driven more by social connection than by individual distress, also supported by contemporary human behaviors research work [29]. Our most significant finding is the robustly protective role of neuroticism (OR 0.29, 95% CI 0.26-0.31) against engaging in SU communities, a direct challenge to the canonical self-medication hypothesis [43]. Instead of reflecting private turmoil, participation in these online discussions was most strongly predicted by the prosocial traits of agreeableness (OR 4.04, 95% CI 3.71-4.41) and extraversion (OR 3.22, 95% CI 2.98-3.49). These results suggest that on public platforms such as Twitter, especially during a crisis such as the COVID-19 pandemic, SU-related conversation often functions as a form of social bonding and affiliation rather than as a direct expression of negative affect.

Overall, this study provides a foundational basis for policy makers and public health practitioners to better understand these dynamics and to design informed, socially aware strategies for addressing SU in online spaces.

## Supplementary material

10.2196/79454 Checklist 1 STROBE Checklist

## Abbreviations

HDBSCAN: Hierarchical Density-Based Spatial Clustering of Applications With Noise
NLP: natural language processing
OR: odds ratio
RQ: research question
STROBE: Strengthening the Reporting of Observational Studies in Epidemiology
SU: substance use
SUD: substance use disorder
UMAP: Uniform Manifold Approximation and Projection
