# Supplementary material for: Intersection of Big Five Personality Traits and Substance Use on Social Media Discourse: AI-Powered Observational Study
Source: J Med Internet Res. 2025 Dec 19;27:e79454. doi: 10.2196/79454 (PMC12716855; doi:10.2196/79454)
Supplement: Checklist 1 [file jmir-v27-e79454-s001.pdf]

Item No.	Section	Item	Location in Manuscript
1	Title/Abstract	Indicate the study's design with a commonly used term in the title or the abstract	Title and Abstract
2	Introduction	Explain the scientific background and rationale for the investigation being reported	Introduction (Paragraph 1-2)
3	Objectives	State specific objectives, including any prespecified hypotheses	Introduction (Last Paragraph)
4	Study Design	Present key elements of study design early in the manuscript	Methods
5	Setting	Describe the setting, locations, and relevant dates, including periods of recruitment, exposure, follow-up, and data collection	Methods (Data Collection)
6	Participants	Cohort study - give the eligibility criteria, and the sources and methods of selection of participants; describe methods of follow-up<br>Case-control study - give the eligibility criteria, and the sources and methods of case ascertainment and control selection; give the rationale for the choice of cases and controls Cross-sectional study - give the eligibility criteria, and the sources and methods of selection of participants Cohort study - for matched studies, give matching criteria and number of exposed and unexposed Case-control study - for matched studies, give matching criteria and the number of controls per case	There is no participant.

7	Variable	Clearly define all outcomes, exposures, predictors, and potential confounders	Methods (Score for Personality Trait, Substance Types, Demographics (age, gender) and other variables like emotions)
8	Data Sources	Describe data sources/measurement tools	Methods (Data Acquision and Substance Use Identification)

9	Bias	Discuss any efforts to address potential sources of bias	Discussions (Limitations)
10	Study size	Explain how the sample size was determined	Methods (Data acquisition and Substance Use Identification)
11	Quantitative Variables	Explain how quantitative variables were handled in the analyses	N/A
12	Statistical methods	Describe all statistical methods, including subgroup analyses and adjustments	Results (Trend Analysis, Biserial Correlation Analysis, Multi Level Logistic Analysis, BERTopic Analysis)
13	Participants	Report the numbers of participants at each stage of the study	No Participation
14	Descriptive Data	Provide summary data for all variables of interest	Results (Descriptive and Trend Analysis in Personality Traits)
15	Outcome Data	Report numbers of outcome events or summary measures	Methods (Demographic and, Emotion Feature Extraction , Big Five Personality Traits Identification)

16	Main Results	Give unadjusted estimates and, if applicable, adjusted estimates and their precision	Results (Paragraph 1)
17	Other Analyses	Report other analyses performed (e.g., subgroup analyses, interactions, sensitivity analyses)	Results (all)
18	Key Results	Summarize key results with reference to study objectives	Results (Personality Traits as Predictors of Substance Use)
19	Limitations	Discuss study limitations, addressing sources of potential bias or imprecision	Discussions (Limitations)
20	Interpretation	Provide interpretation of results, considering objectives, limitations, and other studies	Discussions (Principal Findings)
21	Generalizabilit y	Discuss the generalizability of the study findings	Conclusions (Theoretical and Practical Implications, Policy Implications)
22	Funding	Give the source of funding and the role of funders in the study	Acknowledgement section
